# Essential Oils from *Neolamarckia cadamba*: Methyl Salicylate-Rich Stem Bark Oil as a Multi-Functional Biopesticide with Insecticidal and Antifungal Efficacy

**DOI:** 10.3390/plants14233633

**Published:** 2025-11-28

**Authors:** Han Yao, Yaqian Liu, Xiaohui Liu, Jinyu Zhou, Qianlong Deng, Jiguang Huang

**Affiliations:** State Key Laboratory of Green Pesticide, South China Agricultural University, Guangzhou 510642, China

**Keywords:** *Neolamarckia cadamba* (Roxb.) Bosser, *Anthocephalus cadamba* Miq., essential oil, antifungal activity, mosquitocidal, physiological and biochemical effects, methyl salicylate, biopesticide

## Abstract

The escalating challenges of insecticide resistance and environmental pollution underscore the urgent need for sustainable and multi-functional biopesticides. This study reveals the chemical diversity and potent bioactivity of essential oils (EOs) from *Neolamarckia cadamba*, highlighting their potential as a valuable source of bioactive agents. Gas chromatography–mass spectrometry analysis revealed a striking contrast between the essential oils: the stem bark EO was dominated by methyl salicylate (MeSA, 97.61%), representing the first report of MeSA as a major constituent in this species, while the leaf oil exhibited a complex profile enriched with diterpenoids (25.09%) and fatty acids (23.21%). Both EOs exhibited significant insecticidal efficacy against *Aedes aegypti*, demonstrating rapid knockdown with median knockdown times (KT_50_) of 1.36–1.97 min—surpassing the synthetic dimefluthrin. Additionally, they demonstrated pronounced toxicity, with median lethal concentrations (LC_50_) of 73.41–75.27 μg/mL and fumigant toxicity values of 0.20–0.22 μL/L. Notably, the major component MeSA in the stem bark EO demonstrated obvious insecticidal potential, exhibiting rapid knockdown activity (KT_50_ of 2.29 min), fumigant toxicity (LC_50_ of 1.55 μL/L, 5 h), and poisonous activity (LC_50_ of 92.67 μg/mL, 24 h). Meanwhile, both the stem bark EO and MeSA exhibited strong antifungal activity against the phytopathogen *Rhizoctonia solani*, with median effective concentration (EC_50_) values of 48.70 and 53.91 μg/mL, respectively. This efficacy surpassed that of the commercial fungicide physcion (EC_50_ of 93.34 μg/mL). Additionally, the EOs demonstrated moderate antioxidant activity in the 2,2-diphenyl-1-picrylhydrazyl (DPPH) radical scavenging assay. Mechanistic investigations revealed that the antifungal action of MeSA involved severe cellular disruption, including ultrastructural damage, membrane peroxidation, and critical metabolic suppression via the inhibition of succinate dehydrogenase activity. Our results clearly established *N. cadamba* EOs, particularly the MeSA-rich stem bark oil, as potent, plant-based, and multi-target agent with significant potential for integration into sustainable pest and disease management strategies.

## 1. Introduction

*Neolamarckia cadamba* (Roxb.) Bosser (syn. *Anthocephalus cadamba* Miq.), a tropical tree belonging to the Rubiaceae family, is native to southern Asia and southern China [1]. In ethnomedicine, the bark of *N. cadamba* has traditionally been used to treat inflammation, fever, pruritus, leprosy, dysentery, malaria, and related conditions in China, India, and Southeast Asia [2]. Recognized as a fast-growing “miracle tree”, *N. cadamba* is a valuable resource for construction, furniture manufacturing, and industrial applications such as fiberboard, plywood, and pulp production [3].

Due to its notable pharmacological potential, the bioactive constituents of *N. cadamba* have been extensively studied. To date, various compounds, primarily triterpenoid glycosides, alkaloids, and iridoids, have been identified from its roots, stem bark, leaves, and seeds. Nevertheless, the EO derived from this species remains poorly characterized, despite the well-documented broad-spectrum bioactivities associated with plant EOs, including insecticidal, antimicrobial, herbicidal, anti-inflammatory, and parasiticidal effects [4].

Mosquitoes are among the most important vectors of infectious diseases, transmitting yellow fever, malaria, and Zika virus infections [5,6]. The growing challenges of insecticide resistance and environmental concerns underscore the urgency for sustainable alternatives. Botanical insecticides, particularly those derived from EOs, offer several advantages including low environmental persistence, target-specific modes of action, and a reduced risk of resistance development [7]. Similarly, plant fungal pathogens including *Rhizoctonia solani* Kühn, *Colletotrichum gloeosporioides* Penz., *Fusarium oxysporum* f. sp. *Cubense* (Foc), and *Pyricularia grisea* Sacc cause devastating agricultural losses worldwide. Although conventional synthetic fungicides are effective, they are increasingly associated with resistance development, ecological harm, and non-target toxicity. Hence, plant-based antimicrobials, especially EOs, are increasingly recognized as eco-friendly alternatives [8].

Based on the traditional use and phytochemical richness of *N. cadamba*, and inspired particularly by our field observations of its mosquito-free groves, we hypothesized that its EOs possess significant and previously unexplored pesticidal potential.

In this work, the chemical constituents and bioactivities of bark- and leaf-derived EOs from *N. cadamba* were analyzed. Then, the insecticidal, antifungal, and antibacterial activities were evaluated, and the physiological and biochemical mechanisms of the main active components were further investigated. This investigation highlights the bioactive potential of *N. cadamba* essential oils, providing evidence for their application in next-generation plant-based pesticides and public health strategies against resistant pathogens and vectors.

## 2. Materials and Methods

### 2.1. Plant Material

Fresh stem bark and leaves of *N. cadamba* (approx. 20 m in height) were collected on the campus of South China Agricultural University (Guangzhou, Guangdong province, China) in December 2019 and the identified voucher specimen was deposited in the State Key Laboratory of Green Pesticides, South China Agricultural University, China.

### 2.2. Extraction of Essential Oil

Fresh stem bark (130 g) and leaves of *N. cadamba* (155 g) were separately subjected to hydrodistillation for 4 h using a Clevenger apparatus. The resulting oils were dried over anhydrous sodium sulfate (purity ≥ 99%, Sigma-Aldrich, Darmstadt, Germany). This process yielded 0.7 mL of stem bark EO and 0.15 mL leaf EO, corresponding to yield rates of 0.54% (*v*/*w*) and 0.096% (*v*/*w*), respectively. The EOs were then stored in sealed, dark glass vials at 4 °C until further analysis [9].

### 2.3. Analysis of the Essential Oil

Essential oil analysis was performed using an Agilent Technologies 7693A Gas Chromatograph coupled with a 5977B Mass Spectrometer. A DB-5 MS capillary column (40 m × 0.25 mm × 0.25 μm; Agilent Technologies Inc., Santa Clara, CA, USA) was employed for compound separation. Analyses were conducted using helium as the carrier gas at a flow rate of 1.0 mL/min with a split ratio of 15:1. The oven temperature was programmed as follows: initially held at 40 °C, then increased to 150 °C at a rate of 6 °C/min; further raised to 270 °C at 3 °C/min; and finally increased to 300 °C at 10 °C/min, where it was held for 3 min. The injector and detector temperatures were maintained at 325 °C. A mixture of normal alkanes (C_7_–C_30_, 1000 μg/mL) and essential oil samples (0.8 μL), dissolved in hexane, were injected for analysis. All samples were filtered through a 0.22 μm organic phase filter (BS-QT-013, Biosharp, Haimen, China) prior to injection. The Mass spectra were acquired by electron ionization (EI) at 70 eV, using a spectral range of 30–550 AMU in full scan mode. The MS transfer line (Agilent Technologies Inc., Santa Clara, CA, USA) was maintained at 250 °C [10].

### 2.4. Identification of the Essential Oil Chemical Constituents

Essential oil constituents were identified by comparing their mass spectra with entries in the National Institute of Standards and Technology (NIST 17) Mass Spectral Database or with those of authentic reference compounds. Identification was further confirmed by comparing retention indices with values reported in the literature. The relative percentage of each component in the essential oil was calculated by the area normalization method. The retention index was determined by the following equation:

RI = 100n + 100(t_x_ − t_n_)/(t_n+1_ − t_n_)

where t_n_, t_n+1_, and t_x_ were net retention times.

Identification of the individual components was performed based on the following criteria: (1) comparison of the mass spectra with those of authentic reference compounds, when available, and with spectral data from the NIST 17 database and the Adams terpene library; and (2) comparison of retention indices (RI), determined on an HP-5 column relative to the retention times of a series of n-alkanes (C_7_–C_30_) using linear interpolation, with those of authentic standards or values reported in the literature [10].

### 2.5. Determination of Larvicidal Activity of the Stem Bark Essential Oil and Its Major Constituent MeSA Against Aedes aegypti

Larvicidal activity against *A. aegypti* was evaluated using both the EOs and MeSA as test agents. *A. aegypti* larvae, obtained from the Guangzhou Center for Disease Control and Prevention, were reared in the laboratory for over 10 generations maintained at 26 ± 1 °C, 50 ± 10% relative humidity, and a 16:8 h light-to-dark photoperiod. Early fourth-instar larvae were used for the bioassay. Stock solutions of the test samples were prepared in dimethyl sulfoxide (DMSO, purity ≥ 99%, USP, USA) and subsequently diluted with 0.1% Tween-80 (Sigma-Aldrich, Darmstadt, Germany) in distilled water to obtain a series of final test concentrations: 200, 100, 50, 25, and 12.5 μg/mL. The larvicidal activity of the test samples against *A. aegypti* larvae was determined using the World Health Organization (WHO) recommended liquid immersion method [11]. The treated beakers were placed in an artificial climate chamber maintained at 26 ± 1 °C, 50 ± 10% relative humidity, and a 16:8 h light-to-dark photoperiod. Larval mortality was recorded at 24 and 48 h after the treatment.

The fumigant activity of the EOs of *N. cadamba* and MeSA against *A. aegypti* was determined using a conical flask fumigation method. Twenty 3-day-old healthy female *A. aegypti* mosquitoes were introduced into a 330 mL conical flask. A filter paper strip (3 × 1 cm) was fixed at one end to an absorbent cotton plug using adhesive tape, ensuring complete adhesion to avoid contact with mosquitoes. A volume of 2 μL of the test sample was then applied to the filter paper strip using a micropipette. The flask was immediately sealed with the cotton plug and plastic wrap. Mosquito knockdown status was recorded every 30 s, with knockdown defined as the inability to maintain an upright position or fly normally [12]. The median knockdown time (KT_50_) was calculated based on knockdown rates at different time intervals.

The fumigant lethal activity against adult *A. aegypti* was evaluated using the conical flask fumigation method. *N. cadamba* essential oils (stem bark and leaf) and MeSA were separately diluted in ethanol (≥99.5%, Sigma-Aldrich, Darmstadt, Germany) to generate specific concentration gradients: 0.0625, 0.125, 0.25, 0.5, and 1 μL/L for the essential oils, and 0.25, 0.5, 1, 2, and 4 μL/L for MeSA. For each replicate, a volume of 2 μL of the diluted solution was applied to a filter paper strip (3 × 1 cm) placed inside a 330 mL conical flask. Twenty 3-day-old healthy *A. aegypti* mosquitoes were then introduced into the flask, which was immediately sealed with an absorbent cotton plug and parafilm to initiate a 5 h fumigation period. After exposure, all mosquitoes were transferred to a clean recovery flask containing a 5% glucose solution (glucose, purity ≥ 99%, BioFroxx, Einhausen, Germany) and maintained in an artificial climate chamber at 26 ± 1 °C, 50 ± 10% relative humidity, under a 16:8 h (L:D) photoperiod. Mortality was assessed 24 h after the transfer. A mosquito was considered dead if it showed no response when its abdomen was gently stimulated with a brush [13]. A solvent control (2 μL of ethanol) and an untreated blank control were included.

### 2.6. Determination of Antioxidant Activity

The antioxidant activity was measured following the method described by Abd El-Gawad (2016) [14]. The *N. cadamba* essential oil was tested for radical scavenging activity, using the stable radical 2,2-diphenyl-1-picrylhydrazyl (DPPH, purity ≥ 98%, Sigma-Aldrich, Darmstadt, Germany). A reaction mixture of 1 mL of a hexane solution of the essential oil with different concentrations and equal volume of the ethanol solution of 0.3 mM DPPH was prepared, mixed well, and incubated in the dark for 15 min at room temperature. Ascorbic acid was used as the reference. The decrease in absorbance at 517 nm was determined using a spectrophotometer (UV-8500PC, Metash, Shanghai, China). The IC_50_ (the amount of sample necessary to decrease the absorbance of DPPH by 50%) was calculated. The percentage inhibition of the DPPH radical was calculated using the equation:

inhibition (%) =1 − (Absorbance of sample/Absorbance of control) × 100.

### 2.7. Determination of Antifungal Activity of the Essential Oils and MeSA on Mycelial Growth

The in vitro antifungal activity of the EOs and MeSA on mycelial growth was evaluated against four phytopathogenic fungi *R*. *solani*, *C. gloeosporioides*, *F. oxysporum* f. sp. *cubense*, and *P. grisea*. These fungal isolates were originally obtained from symptomatic rice, pepper, and banana plants at the Teaching and Research Base of South China Agricultural University. Following isolation and identification, the cultures were preserved in the Department of Plant Pathology, South China Agricultural University. Antifungal assays were conducted using a modified method from Kamaruzzaman et al. [15]. Stock solutions of the test samples and the positive control (physcion, purity ≥ 98.0%, Merck, Darmstadt, Germany) were prepared by dissolving 0.1 g of each compound in DMSO and diluting to a final volume of 1 mL, resulting in a concentration of 100,000 μg/mL. Then, 5 mL of potato dextrose agar (PDA) medium was dispensed into 70 mm sterile Petri dishes. After cooling to approximately 40 °C, 25 μL of the stock solution was added to the medium and thoroughly mixed to ensure uniform distribution. This procedure was repeated with appropriate dilutions of the stock solution to obtain the following final concentrations: 120, 60, 30, 15, and 7.5 μg/mL for *R. solani*; 1000, 500, 250, 125, and 62.5 μg/mL for *C. gloeosporioides*; 2000, 1000, 500, 250, and 125 μg/mL for both *F. oxysporum* f. sp. *cubense* and *P. grisea*. A solvent control containing 0.5% DMSO (*v*/*v*) and a blank control containing 0.5% sterile water (*v*/*v*) were included in each experiment. A 6 mm diameter agar plug, taken from the margin of an actively growing fungal culture, was placed in the center of the solidified medium. Plates were sealed and incubated in an artificial climate chamber at 26 ± 1 °C, 50 ± 10% relative humidity, under 16:8 h (L:D) photoperiod until the mycelial growth in the control plates reached approximately 80% of the plate diameter. Mycelial diameter was then measured using the cross method [16].

### 2.8. Physiological and Biochemical Effects of MeSA on R. solani

#### 2.8.1. Hyphal Morphology of *R. solani*

The effect of MeSA on *R. solani* hyphal morphology was assessed in vitro. MeSA was added to PDA medium at concentrations of 0, 12.5, 25, 50, 100, and 200 μg/mL. A 5 mm agar plug from an actively growing *R. solani* culture was then inoculated at the center of each treatment plate, which was then incubated upside down at 25 °C. The control group was treated with DMSO at the same concentration used in the MeSA treatments. After incubation, hyphae from the surface of the culture medium were carefully scraped, suspended in distilled water on the glass slide, and examined under light microscopy (Leica DMLB2, Leica Microsystems, Wetzlar, Germany) to observe morphological changes induced by different MeSA treatments. Transmission electron microscopy (TEM) analysis was conducted using a FEI Tecnai 12 instrument (FEI Company, Hillsboro, CA, USA) to examine the effect of MeSA at 50 µg/mL on the ultrastructure of *R. solani*. The TEM samples were processed as described previously by Houot et al. [17].

#### 2.8.2. Weight and Quantity of Sclerotium of *R. solani*

*R. solani* cultures were incubated on PDA medium at 25 °C for 10 days in the presence of MeSA at concentrations ranging from 12.5 to 200 μg/mL. Cultures treated with DMSO served as the control. After incubation, sclerotia were carefully harvested from the culture medium, transferred into 2 mL microcentrifuge tubes, and counted. The dry weight of the sclerotia was then determined after drying in an oven (DHG-9240, JingHong, Shanghai, China) at 60 °C until a constant weight was achieved.

#### 2.8.3. Detection of Soluble Protein

Soluble protein content was determined using the Bradford assay, with bovine serum albumin (BSA, purity ≥ 98.0%, Sigma-Aldrich, Darmstadt, Germany) as the standard. A BSA stock solution (1000 μg/mL) was prepared, and serial dilutions were made to obtain BSA standard solutions at concentrations of 0, 125, 250, 375, 500, 625, 750, and 1000 μg/mL. The Coomassie Brilliant Blue G-250 reagent (CBB G-250, purity ≥ 95%, Sigma-Aldrich, Darmstadt, Germany) was prepared by dissolving 0.01 g of CBB G-250 in 5 mL of 95% ethanol, followed by the addition of 10 mL of 80% phosphoric acid. The solution was then diluted to 100 mL with deionized water and filtered. To generate the standard curve, 0.1 mL of each BSA standard solution was mixed with 5 mL of the CBB G-250. After a 5 min incubation, the absorbance was measured at 595 nm using a spectrophotometer. *R. solani* sclerotia treated with MeSA were cultured at 25 °C for 10 days, with DMSO-treated cultures serving as control. After incubation, 0.2 g of sclerotia was homogenized in 1.5 mL of phosphate buffer (pH 7.4; Sigma-Aldrich, Darmstadt, Germany) using a mortar and pestle under frozen conditions. The homogenate was then transferred to a 10 mL centrifuge tube and centrifuged at 4000 rpm for 10 min. The supernatant was collected for protein quantification. A 0.1 mL aliquot of the supernatant was then mixed with 5 mL of CBB G-250, incubated for 5 min, and the absorbance was measured at 595 nm. Soluble protein concentrations in the samples were then calculated using the BSA standard curve. The soluble protein content was determined using the following formula:

Soluble protein content (mg/g) = soluble protein concentration (μg/mL) × total volume of extract (mL)/(sample volume for analysis (mL) × sample weight (g) × 1000)

#### 2.8.4. Detection of Malondialdehyde (MDA) Content

MDA content, an indicator of lipid peroxidation, was determined using the thiobarbituric acid (TBA) method. *R. solani* mycelia (0.5 g) treated with MeSA for 10 days were homogenized in 5 mL of 10% trichloroacetic acid (TCA, purity ≥ 99%, Sigma-Aldrich, Darmstadt, Germany) solution using a mortar and pestle. The homogenate was then centrifuged at 4000 g for 15 min. A 2 mL aliquot of the supernatant was mixed with 2 mL of 0.6% TBA solution. The mixture was heated in a water bath at 100 °C for 20 min. After cooling to room temperature, the absorbance was measured at 532, 600, and 450 nm using a spectrophotometer.

MDA concentration: C (mmol/L) = 6.45 × (OD_532_ − OD_600_) − 0.56 × OD_450_

MDA content = MDA concentration (mmol/L) × Total volume of extract (L)/sample (g).

#### 2.8.5. Detection of Cell Membrane Permeability

The effect of MeSA on *R. solani* cell membrane permeability was assessed by measuring relative electrical conductivity [18]. *R. solani* was cultured in PDA medium. Five agar plugs of *R. solani* (untreated with MeSA) were inoculated into 100 mL of PDA medium and incubated on a rotary shaker (THZ-300C, Yiheng, Shanghai, China) at 25 °C and 120 rpm for 24 h. Subsequently, the cultures were treated with different concentrations of MeSA (5 mL per flask) and further incubated for 12 h. Hyphae were then collected by filtration using filter paper, washed, and dried. The dried hyphae were subsequently ground under frozen conditions. The resulting material was then transferred to a 25 mL centrifuge tube, and the volume was adjusted to 20 mL with distilled water. After 20 min’s gentle stirring, the electrical conductivity (R_1_) of the suspension was measured using a conductivity meter (FE32-Meter, Mettler toledo, Shanghai, China). To determine the total conductivity, the suspension was heated in boiling water for 15 min to disrupt the cells, thereby killing the pathogen. After cooling the suspension to room temperature by placing the tube under running water for 10 min, the electrical conductivity (R_2_) was measured. Relative conductivity was calculated as the ratio of R_1_ to R_2_ (R_1_/R_2_) [19].

#### 2.8.6. Succinate Dehydrogenase (SDH) Activity Assay

SDH activity was determined using a commercially available SDH assay kit (MAK561, Sigma-Aldrich, Darmstadt, Germany). Fresh *R. solani* mycelia (0.1 g) treated with MeSA for 8 days were homogenized in reagent 1 (1 mL) from the assay kit, followed by the addition of 10 μL of reagent 2. Homogenization was performed in an ice bath. The homogenate was then centrifuged at 11,000× *g* for 15 min at 4 °C. Following centrifugation (TGL-16M, Cence, Changsha, China), the supernatant was collected and kept on ice. Then, for the assay, a reaction mixture was prepared containing 168 μL of Reagent 3, 12 μL of Reagent 5, and 20 μL of the supernatant. Absorbance at 600 nm was measured at 20 s (A_1_) and 80 s (A_2_) after initiating the reaction, and the change in absorbance (ΔA = A_2_ − A_1_) was calculated.

SDH (U·mg^−1^) = (ΔA × V_total reaction_ × V_total sample_ × 10^9^)/(ε × d × W × V_sample_ × T)

Note: V_total reaction_: 2 × 10^−4^ L; ε: the extinction coefficient of 2, 6-dichloroindophenol, 2.1 × 10^4^ L/mol/cm; d: the light diameter of the cuvette, 1 cm; V_total sample_: 2 × 10^−5^ L; W: weight of sample, 0.1 g; T: reaction time, 60 s.

### 2.9. Statistical Analysis

Data were analyzed using IBM SPSS Statistics, Version 19.0 (International Business Machines Corporation, Armonk, NY, USA). Analysis of variance (ANOVA) was used to determine significant differences among treatments (*p* < 0.05), followed by Duncan’s multiple range test or Student’s *t*-test for pairwise comparisons. Median effective concentrations (EC_50_) and median inhibitory concentrations (IC_50_), with 95% confidence intervals, were calculated by probit analysis. Each treatment included three replicates. All experiments were repeated at least three times. All quantitative data were presented as the mean ± standard deviation (SD) of at least three independent experiments.

## 3. Results

### 3.1. Chemical Components Identified in the Essential Oil of the Stem Barks

**Figure 1:**
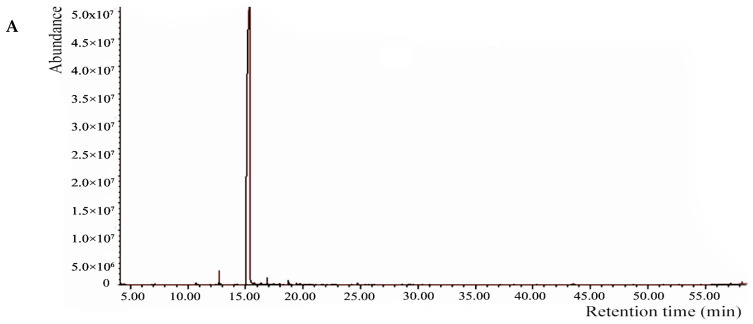

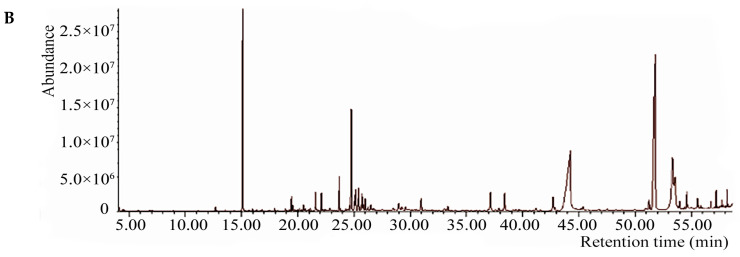
GC-MS chromatograms of essential oils from *N. cadamba*: (**A**) Stem bark EO; (**B**) Leaf EO.

The components of the essential oil of stem bark of *N. cadamba* are listed in Table 1. The GC-MS chromatogram of the essential oil is shown in Figure 1A. A total of 17 components were identified accounting for 99.73% of the total oil composition. MeSA was the most abundant compound, accounting for 97.61% of the total oil. Minor constituents included linalool (0.58%), ethyl salicylate (0.30%), *n*-hexadecanoic acid (0.19%), chavibetol (0.19%), and nerolidol (0.12%). All other components were present at levels below 0.10% (Table 1).

### 3.2. Chemical Components Identified in the Essential Oil of the Leaves

The GC-MS chromatogram (Figure 1B) and detailed composition (Table 2 and Table S1) of *N. cadamba* leaf essential oil revealed a complex profile of 132 identified constituents, accounting for 93.94% of the total oil. Compared to the stem bark EO, the leaf EO was characterized by a greater diversity of compound classes. The major compound classes identified in the leaf EO included diterpenoids (25.09%), fatty acids (23.21%), esters (18.64%), and sesquiterpenoids (11.33%). Table 2 lists the 18 individual components with relative contents exceeding 0.5%, while the complete list of 132 identified constituents is provided in Table S1.

### 3.3. Poisonous, Knockdown, and Fumigant Activities of N. cadamba Stem Bark Essential Oil and Its Main Component, MeSA, Against A. aegypti

The poisonous activity of *N. cadamba* essential oils was evaluated against the 4th instar larvae of *A. aegypti* (Table 3). The LC_50_ values (24 h) for the stem bark and leaf essential oils were 75.27 μg/mL and 73.41 μg/mL, respectively. MeSA, the major component of the stem bark oil, also demonstrated poisonous activity, with an LC_50_ value of 92.67 μg/mL after 24 h.

Knockdown assays demonstrated that the stem bark essential oil, leaf essential oil, and MeSA all possessed significant knockdown activity against the adult female *A. aegypti*, with KT_50_ values of 1.97, 1.36, and 2.29 min, respectively. In contrast, the KT_50_ value for the commercial pyrethroid insecticide dimefluthrin was 2.48 min (Table 3), indicating that both the stem bark and leaf essential oils, as well as MeSA, exhibited faster knockdown activity against the adult female *Aedes aegypti*.

Furthermore, the stem bark essential oil, leaf essential oil, and MeSA exhibited significant fumigant activity 5 h after treatment, with LC_50_ values of 0.22, 0.20, and 1.55 μL/L, respectively (Table 3).

**Table 1:** Chemical components identified in *N. cadamba* stem bark essential oil by GC-MS.

No.	RT	Compounds	Molecular Formula	Percentage (%)	RI_a_	RI_b_	RI_c_	Class	Match(%)	CAS
1	15.39	Methyl salicylate	C_8_H_8_O_3_	97.61	1209	1194 [20]	1192	Ester	98.39	119-36-8
2	12.69	Linalool	C_10_H_18_O	0.58	1099	1097 [20]	1099	Monoterpene Alcohol	99.39	78-70-6
3	16.87	Ethyl salicylate	C_9_H_10_O_3_	0.30	1272	1193 [21]	1269	Ester	97.55	118-61-6
4	18.74	m-Eugenol	C_10_H_12_O_2_	0.19	1351	1370 [22]	1375	Phenylpropanoid	96.73	501-19-9
5	43.55	n-Hexadecanoic acid	C_16_H_32_O_2_	0.19	1957	1961 [23]	1968	Fatty Acids	92.66	1957-10-3
6	24.73	Nerolidol	C_15_H_26_O	0.12	1557	2053 [24]	1564	Sesquiterpene Alcohol	97.52	7212-44-4
7	16.35	Geraniol	C_10_H_18_O	0.08	1250	1254 [20]	1255	Monoterpene Alcohol	96.41	106-24-1
8	10.69	3-Ethyl-4-methylpentan-1-ol	C_8_H_18_O	0.07	1019	1020 [25]	1023	Alcohol	96.97	38514-13-5
9	12.81	Nonanal	C_9_H_18_O	0.07	1104	1103 [20]	1104	Aldehyde	97.36	124-19-6
10	14.22	(*E*)-2-Nonenal	C_9_H_16_O	0.07	1161	1164 [23]	1162	Aldehyde	95.66	18829-56-6
11	19.43	*β*-Damascenone	C_13_H_18_O	0.07	1380	1385 [23]	1386	Ketone	93.94	23726-93-4
12	19.74	*β*-Elemene	C_15_H_24_	0.07	1394	1391 [26]	1391	Sesquiterpene Hydrocarbon	92.95	515-13-9
13	7.11	1-Hexanol	C_6_H_14_O	0.06	864	864 [23]	868	Alcohol	95.46	111-27-3
14	17.42	Dihydroedulane II	C_13_H_22_O	0.06	1295	2089 [27]	1318	Cyclic Ether	89.91	41678-32-4
15	17.97	(*E,E*)-2,4-Decadienal	C_10_H_16_O	0.06	1318	1315 [28]	1317	Aldehyde	92.54	25152-84-5
16	29.31	N-Hexyl salicylate	C_13_H_18_O_3_	0.06	1674	1683 [29]	1683	Ester	90.36	6259-76-3
17	58.21	2,2’-Methylenebis (6-tert- butyl-4-methyl-phenol)	C_23_H_32_O_2_	0.06	2398	-	2414	Phenol	75.48	119-47-1
			Total	99.73						

Note: RT, the retention time; RI_a_, the Kovats retention index of each component calculated by NIST software (2.3) and ion spectrum of C7-C30 n-alkane mixture; RI_b_, the retention index in NIST 17 mass spectrometry library; RI_c_, the retention index in the literature.

**Table 2:** Major chemical constituents (≥ 0.5% in content) of the essential oil from *N. cadamba* leaves identified by GC-MS.

No.	RT	Compounds	Molecular Formula	Percentage (%)	RI_a_	RI_b_	RI_c_	Class	Match	CAS
1	51.78	Phytol	C_20_H_40_O	23.32	2108	2104 [24]	2114	Diterpene	98.81	150-86-7
2	44.25	n-Hexadecanoic acid	C_16_H_32_O_2_	18.41	1970	1961 [23]	1968	Fatty acid	97.66	1957/10/3
3	15.10	Methyl salicylate	C_8_H_8_O_3_	9.83	1197	1194 [20]	1192	Ester	99.48	119-36-8
4	53.33	1-Heneicosene	C_21_H_42_	8.18	2145	2096 [28]	2089.1	Alkene	70.90	1599-68-4
5	24.77	Nerolidol	C_15_H_26_O	5.33	1559	2053 [25]	1564	Sesquiterpene Alcohol	98.50	7212-44-4
6	53.56	Linolenic acid	C_18_H_30_O_2_	4.61	2150	2020 [30]	2139	Fatty acid	93.71	463-40-1
7	23.70	(*-*)-Spathulenol	C_15_H_24_O	1.61	1528	1599 [10]	1577	Sesquiterpene Alcohol	81.75	77171-55-2
8	38.39	Benzyl salicylate	C_14_H_12_O_3_	1.44	1861	1790 [31]	1869	Ester	96.70	118-58-1
9	25.15	(*Z*)-3-Hexenyl benzoate	C_13_H_16_O_2_	1.42	1569	2148 [32]	1570	Ester	97.30	25152-85-6
10	37.14	Hexahydrofarnesyl acetone	C_18_H_36_O	1.41	1837	1848 [33]	1844	Ketone	97.20	502-69-2
11	25.71	(*E*)-2-Hexenyl benzoate	C_13_H_16_O_2_	1.27	1585	2182 [34]	1588	Ester	82.30	76841-70-8
12	25.42	Benzoic acid, hexyl ester	C_13_H_18_O_2_	1.19	1577	1576 [30]	1580	Ester	98.13	6789-88-4
13	54.60	Ethyl linolenate	C_20_H_34_O_2_	1.16	2175	2073 [30]	2169	Ester	88.87	1191-41-9
14	42.70	Isophytol	C_20_H_40_O	1.12	1942	1939 [28]	1948	Diterpene Alcohol	97.22	505-32-8
15	30.97	1,2-Epoxyhexadecene	C_16_H_32_O	0.82	1712	-	1708	Epoxide	89.10	7320-37-8
16	21.58	Cabreuva oxide B	C_15_H_24_O	0.72	1460	1458 [21]	1465	Sesquiterpene Epoxide	95.92	107602-53-9
17	26.00	(*E*)-*β*-Farnesene epoxide	C_15_H_24_O	0.64	1594	1624 [35]	1624	Sesquiterpene Epoxide	84.60	83637-40-5
18	53.96	Phytol, acetate	C_22_H_42_O_2_	0.59	2159	2215 [33]	2168	Ester	86.20	10236-16-5
			Total	83.04						

Note: RT, the retention time; RI_a_, the Kovats retention index of each component calculated by NIST software (2.3) and ion spectrum of C7-C30 n-alkane mixture; RI_b_, the retention index in NIST 17 mass spectrometry library; RI_c_, the retention index in the literature.

**Table 3:** Poisonous, knockdown, and fumigant activity of *N. cadamba* stem bark essential oil and its main component, MeSA, against *A. aegypti*.

Activity	Treatment	Time	Regression Eq.	LC_50_^+^ (μg/mL) /KT_50_^&^ (min)	Correlation Coefficient (r)	95% Confidence Limit (μg/mL)
Poisonous activity (LC_50_)	EO-ST *	24 h	y = −15.75 + 8.42x	75.27	0.97	57.75–100.88
	EO-L #	24 h	y = −7.39 + 3.96x	73.41	0.97	64.34–84.35
	MeSA	24 h	y = −9.78 + 5.10x	92.67	0.98	60.36–120.03
	EO-ST	48 h	y = 1.35 + 1.99x	67.56	0.99	51.87–88.00
	EO-L	48 h	y = 1.93 + 1.71x	61.97	0.98	45.35–83.53
	MeSA	48 h	y = −9.78 + 5.10x	82.68	0.98	60.36–120.03
Knockdown activity (KT_50_)	EO-ST	-	y = −1.82 + 2.68x	1.97	0.97	1.66–2.28
	EO-L	-	y = −0.79 + 2.56x	1.36	0.99	0.99–1.71
	MeSA	-	y = −1.82 + 2.68x	2.29	0.97	1.66–2.28
	dimefluthrin	-	y = −2.92 + 3.21x	2.48	0.95	2.19–2.82
Fumigant activity (LC_50_)	EO-ST	5 h	y = 6.36 + 2.07x	0.22	0.99	0.17–0.28
	EO-L	5 h	y = 6.23 + 1.78x	0.20	0.98	0.15–0.27
	MeSA	5 h	y = 4.67 + 1.69x	1.55	0.98	1.14–2.11

Note: *: EO of the stem bark; #: EO of the leaves; ^+^: median lethal concentration; ^&^: median knockdown time.

### 3.4. Antioxidant Activity

Given their potential as natural alternatives for synthetic antioxidants in food preservation, essential oils have attracted considerable attention. In this study, the antioxidant activity of *N. cadamba* essential oils was evaluated using the DPPH radical scavenging assay. This assay was based on the reduction in the stable free radical DPPH, which exhibited a deep violet color, to DPPH, a colorless compound, upon reaction with an antioxidant. Free radical scavenging activity was typically expressed either as the percentage of DPPH inhibition or as the concentration of antioxidant required to reduce the DPPH radical concentration by 50% (IC_50_). A lower IC_50_ value indicated a higher antioxidant potency. The results showed that the positive control ascorbic acid exhibited the strongest antioxidant activity with an IC_50_ value of 0.02 mg/mL. In comparison, the IC_50_ values of the essential oils from *N. cadamba* stem bark and leaves were 1.23 and 3.29 mg/mL, respectively (Table 4), indicating a moderate capacity to scavenge DPPH radicals.

### 3.5. Antifungal Activities of N. cadamba Essential Oil and MeSA

The effects of *N. cadamba* stem bark essential oil on the mycelial growth of *R. solani*, *F. oxysporum*, *C. gloeosporioides*, and *P. grisea* are summarized in Table 5. The antifungal activity, as estimated by the EC_50_ values, varied considerably among the fungal species (Figure S1 and Table 5). The lowest EC_50_ value was observed against *R. solani* (48.70 μg/mL), indicating the highest antifungal activity, while the highest EC_50_ value was detected against *F. oxysporum* (1229.48 μg/mL), indicating the lowest antifungal activity. The antifungal activities of MeSA against these four fungal species were also determined (Table 5). The EC_50_ values of MeSA against *R. solani*, *F. oxysporum*, *C. gloeosporioides*, and *P. grisea* were 53.91, 1045.11, 854.17, and 1041.76 μg/mL, respectively. In comparison, the EC_50_ values of physcion against *R. solani* was 93.34 μg/mL. These results indicated the essential oil from the stem bark of *N. cadamba,* and the active ingredient MeSA exhibited notable antifungal activity against *R. solani*.

### 3.6. Effect of MeSA on Sclerotium Weight and Quantity of R. solani

**Figure 2:**
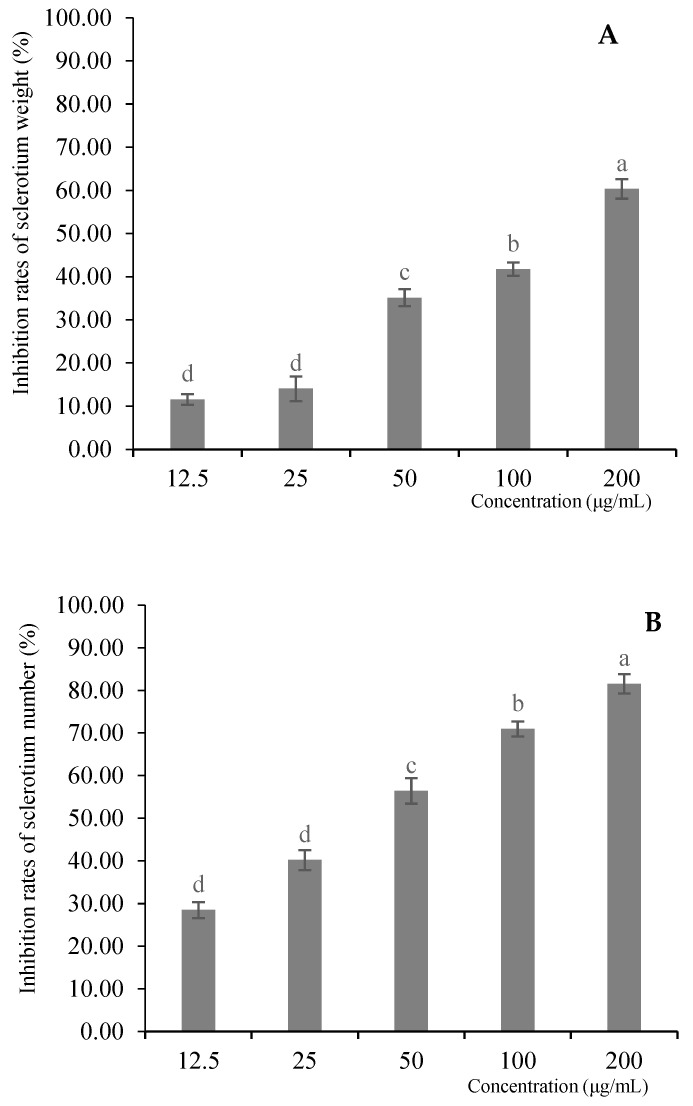
Inhibition of *R. solani* sclerotium: (**A**) weight; (**B**) number by MeSA. Different letters indicated significant differences (*p* < 0.05).

In general, the sclerotium weight was higher in the control group compared to that of the MeSA treated groups. Treatment with MeSA at 200 μg/mL resulted in a 60.33% reduction in sclerotium weight. Similarly, the number of sclerotia formed by *R. solani* decreased with increasing MeSA concentrations. Specifically, treatment with MeSA at 200 μg/mL and 100 μg/mL resulted in inhibition rates of 81.56% and 70.95%, respectively, in sclerotium number. Treatment with MeSA resulted in a concentration-dependent reduction in both the weight and number of sclerotia produced by *R. solani* (Figure 2).

### 3.7. Effect of MeSA on Hyphal Morphology and Ultrastructure of R. solani

#### 3.7.1. Effects of MeSA on Mycelial Morphology and General Ultrastructure of *R. solani*

**Figure 3:**
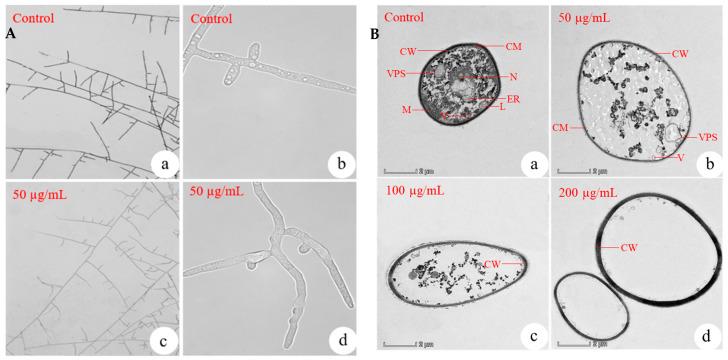
MeSA altered the mycelial morphology and general ultrastructure of *R. solani*. (**A**) Morphology of hyphae under optical microscope. (**a**,**b**) Control hyphae at 5× and 100× magnification, respectively; (**c**,**d**) Hyphae treated with 50 μg/mL MeSA at 5× and 100× magnification. (**B**) General ultrastructure observed by TEM (5300×). (**a**) Control hyphae; (**b**) Hyphae treated with 50 μg/mL MeSA; (**c**,**d**) Hyphae treated with 100 and 200 μg/mL MeSA, respectively. Abbreviations: CW, cell wall; VPS, vesicle production system; M, mitochondria; CM, cell membrane; N, nucleus; ER, endoplasmic reticulum; L, lomasome; V, vesicle.

MeSA treatment significantly altered the mycelial morphology of *R. solani*. Microscopic observation revealed distinct differences between the control and the MeSA-treated hyphae. In the control, the hyphae exhibited smooth surfaces, right-angled branching with prominent barrel-shaped constrictions at the base of each branch (Figure 3A(a,b)).

In contrast, *R. solani* treated with MeSA displayed abnormal mycelial development, characterized by irregular protrusions (Figure 3A(c,d)). The hyphae appeared collapsed and flaccid, with reduced branching angles and a noticeable loss of rigidity and erectness. Furthermore, MeSA treatment induced a clear dose-dependent response in the ultrastructural alterations of fungal cells. In control cells, typical organelles such as vesicles, mitochondria, endoplasmic reticulum, vesicle-producing systems, and lomasomes were clearly visible, along with intact nuclei, cell membranes, and cell walls (Figure 3B(a)). However, MeSA treatment induced progressive cellular damage with increasing concentrations. At 50 µg/mL, partial cell membrane disruption was observed (Figure 3B(b)). At 100 µg/mL, extensive loss of membrane structure occurred (Figure 3B(c)). At 200 µg/mL, structural integrity of organelles and nuclei progressively diminished, with near-complete organelle degradation and an almost complete absence of the cell membrane (Figure 3B(d)).

#### 3.7.2. Effects of MeSA on Mitochondria, Endoplasmic Reticulum, Vesicle Production System and Vesicles in *R. solani*

**Figure 4:**
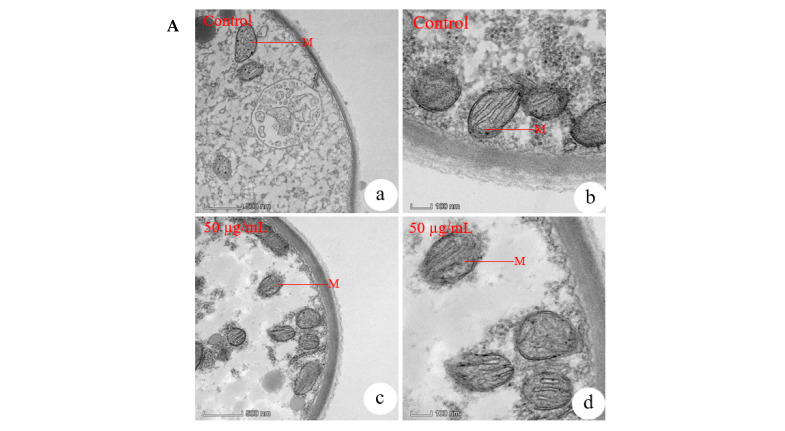

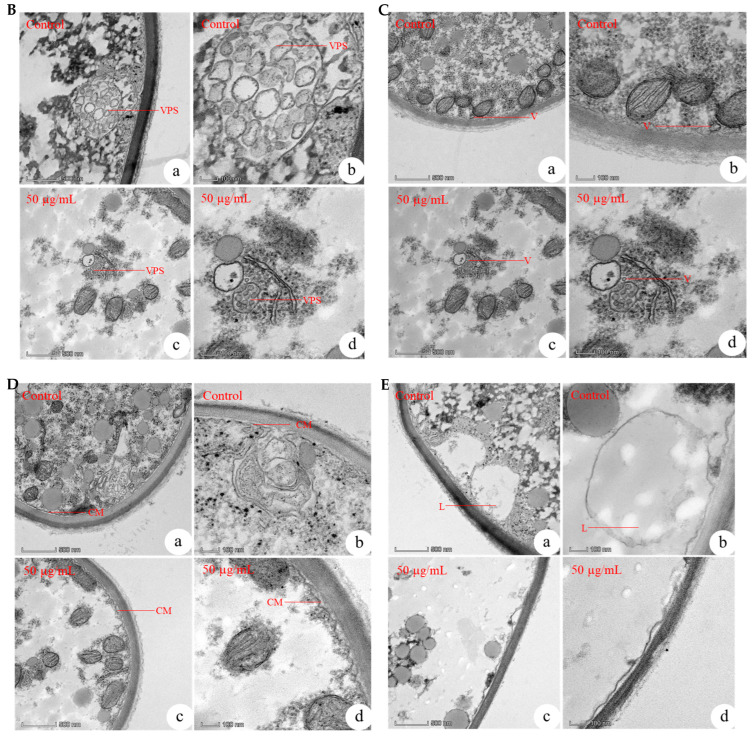
MeSA disrupted mitochondrial, vesicle production system (VPS), vesicle ultrastructure, cell membrane, and lomasome ultrastructure in *R. solani*: (**A**) Mitochondrial ultrastructure. (**a**,**b**) Control mitochondria; (**c**,**d**) Mitochondria treated with 50 μg/mL MeSA. (**B**) Vesicle production system (VPS). (**a**,**b**) Control group; (**c**,**d**) Treated with 50 µg/mL MeSA. (**C**) Vesicle ultrastructure. (**a**,**b**) Control group; (**c**,**d**) Treated with 50 µg/mL MeSA. (**D**) Cell membrane integrity. (**a**,**b**) Control group; (**c**,**d**) Treated with 50 µg/mL MeSA. (**E**) Lomasome ultrastructure. (**a**,**b**) Control group; (**c**,**d**) Treated with 50 µg/mL MeSA. Magnifications: 22,000× (**a**,**c** in all panels) and 57,000× (**b**,**d** in all panels). Abbreviations: M, mitochondria; VPS, vesicle production system; V, vesicle; CM, cell membrane; L, lomasome.

TEM revealed distinct ultrastructural changes in *R. solani* mitochondria following MeSA treatment. In control cells, both mitochondria (Figure 4A(a,b)) exhibited normal, well-defined morphology. However, exposure to 50 µg/mL MeSA induced progressive organelle damages, including structural disruption and a quantitative reduction in mitochondria (Figure 4A(c,d)). These degenerative effects became more pronounced at 100 µg/mL, where a dramatic decrease in organelle numbers was observed (Figure 3B(c)). At the highest concentration (200 µg/mL, Figure 3B(d)), the structural integrity of key organelles was entirely lost. These dose-dependent ultrastructural alterations demonstrated that MeSA could disrupt cellular organelles in *R. solani.*

Our ultrastructural analysis also demonstrated significant, dose-dependent disruptions to the vesicle production system (VPS) (Figure 4B(c,d)) and vesicles (Figure 4C(c,d)) in *R. solani* following MeSA treatment. The vesicular components exhibited progressive structural damage and a quantitative reduction with increasing MeSA concentrations. As it was shown, exposure to 50 µg/mL MeSA resulted in visible structural deterioration and reduced numbers of both the VPS (Figure 4B(c,d)) and vesicles (Figure 4C(c,d)). The damage to the secretory system was particularly severe and initiated at a lower concentration. Complete disintegration of the vesicle production system (VPS) was observed at 100 µg/mL (Figure 3B(c)), and by 200 µg/mL, vesicles were nearly undetectable (Figure 3B(d)).

These findings revealed that MeSA targeted the fungal secretory pathway, with the VPS exhibiting greater sensitivity to the treatment than the vesicles themselves. The sequential disruption of these critical cellular components suggested a specific mode of action for MeSA in impairing fungal transport and secretion mechanisms.

TEM further revealed dose dependent ultrastructural alterations in both the cell membrane and lomasomes of *R. solani* following MeSA treatment. The cell membrane exhibited progressive damage, with initial alterations observed at 50 µg/mL MeSA (Figure 4D(c,d)) and nearly complete disintegration at 200 µg/mL (Figure 3B(d)). In contrast, lomasomes demonstrated greater sensitivity to MeSA, showing complete disappearance even at the lower concentration of 50 µg/mL (Figure 4E(c,d)). These differential responses suggested that MeSA might affect the cell membrane and lomasomes in different ways.

### 3.8. Effects of MeSA on Physiological and Biochemical Parameters of R. solani

**Figure 5:**
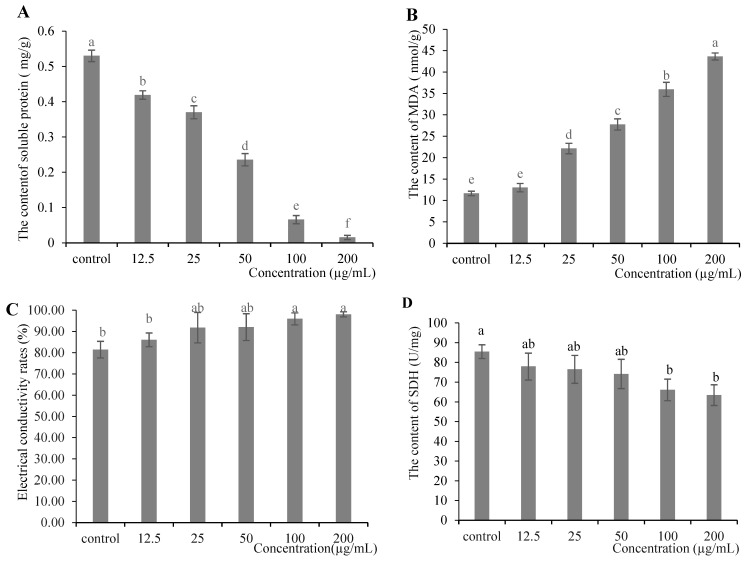
Physiological and biochemical effects of MeSA on *R. solani*: (**A**) Soluble protein content; (**B**) MDA content; (**C**) Relative conductivity; (**D**) SDH activity. Control: DMSO treatment. Different letters indicated significant differences (*p* < 0.05).

The soluble protein content in *R. solani* decreased in a dose-dependent manner from 0.53 to 0.015 mg/g as MeSA treatment increased from 0 to 200 µg/mL (*p* < 0.05). These results indicated that MeSA effectively suppress soluble protein synthesis in the pathogen, thereby inhibiting its growth (Figure 5A).

Lipid peroxidation, measured by MDA content, increased from 13.01 to 43.66 nmol/g in *R. solani* mycelia with increasing MeSA concentrations. The 200 µg/mL MeSA treatment induced a nearly 4-fold increase in MDA content compared to the control, indicating severe membrane damage, and impaired cellular integrity (Figure 5B).

The electrical conductivity of *R. solani* cells increased significantly with increasing concentrations of MeSA (*p* < 0.05). Treatment with 200 µg/mL MeSA resulted in a conductivity rate of 98.07% compared to the control, indicating enhanced electrolyte leakage. These results demonstrated that MeSA disrupted the cell membrane integrity of *R. solani*, leading to increased permeability and electrolyte efflux (Figure 5C).

SDH activity in *R. solani* treated with MeSA ranged from 63.49 to 85.46 U/mg. The 200 µg/mL MeSA treatment resulted in a 34.60% reduction in SDH activity compared to other concentrations, demonstrating a significant inhibitory effect (*p* < 0.05). These findings indicated that MeSA impaired mitochondrial respiration in the pathogen, thereby suppressing its growth (Figure 5D).

## 4. Discussion

Our study presents a comprehensive analysis of the chemical profiles and bioactivities of EOs extracted from different parts of *N. cadamba*, revealing their strong potential as multi-functional biopesticides. The GC-MS analysis revealed a striking chemical difference between the stem bark and leaf EOs. Notably, the stem bark EO was characterized by an exceptionally high concentration of methyl salicylate (MeSA, 97.61%), which, to our knowledge, represented the first report identifying MeSA as the dominant constituent in this species.

In contrast, the leaf EO presented a more complex mixture, rich in diterpenoids and fatty acids, consistent with the greater chemical diversity commonly reported for the leaf of plant [36]. It was noteworthy that the chemical profile of our leaf EO also differed from the previously reported result for *N. cadamba* leave EO using headspace solid-phase microextraction (HS-SPME) [37]. This divergence underscored how analytical methodologies and plant material collectively affected the final chemical profile. Specifically, HS-SPME targeted primarily the headspace volatiles released under specific conditions. Furthermore, the plants used in the previous study were young trees (1.5–2 m in height), in contrast to the mature trees (approx. 20 m in height) employed in our work. It is well-established that secondary metabolite profiles, particularly defense-related compounds, can vary dramatically with plant ontogeny [38,39]. These factors likely accounted for the differences observed even for the same plant organ. In this context, the discovery of the MeSA-dominated stem bark EO of *N. cadamba* was a novel finding.

Furthermore, both essential oils demonstrated notable insecticidal activity against *A. aegypti*, particularly through a rapid knockdown effect that exceeded the efficacy of the synthetic dimefluthrin. Our result also showed that MeSA was the main active ingredient in the stem bark EO. This result was consistent with the known bioactivities of MeSA against various insect pests [36]. Furthermore, the presence of linalool, the second most abundant component in the stem bark oil, might contribute to the overall insecticidal effect, as its activity against *A. aegypti* had been previously documented [40].

Beyond insecticidal activity, a key finding of our work was the pronounced and specific antifungal activity of the stem bark EO and MeSA against *R. solani*. The EC_50_ values for both the oil and MeSA were lower than that of the commercial fungicide physcion, highlighting their potential efficacy. While a previous study reported the inhibition of *R. solani* by MeSA, our research provided deeper mechanistic insights [41]. We found that the antifungal action of MeSA was multifaceted, involving direct cellular damage. Our ultrastructural evidence indicated severe disruption to organelles, including mitochondria and the endoplasmic reticulum, together with the loss of membrane integrity, as confirmed by the increased MDA content and electrolyte leakage. Meanwhile, we also demonstrated the inhibition of SDH activity, suggesting a disruption of the mitochondrial respiratory chain. This combination of membrane damage and suppression of core metabolic energy production likely contributed to the potent antifungal effect observed.

The practical implications of using *N. cadamba* EOs, particularly the MeSA-rich stem bark oil, extend beyond direct toxicity. MeSA was a recognized plant signal molecule involved in defense responses [42].

Therefore, the application of this EO in an agricultural setting could offer a dual benefit. It could act directly against fungal pathogens and insect pests while also potentially priming plant defense mechanisms, as evidenced by studies where MeSA induced resistance in plants [43,44] and enhanced the biological control activity of natural enemies [45]. These multi-faceted effects represented a distinct advantage of plant-derived EOs compared to conventional, single-site synthetic pesticides, potentially mitigating the risk of resistance development [46].

In summary, our results highlighted *N. cadamba* EOs, especially the stem bark oil, as a promising source of eco-friendly biopesticidal agents. We identified the previously unreported MeSA-rich stem bark and elucidated a multi-target antifungal mechanism involving cellular ultrastructural disruption, membrane damage, and metabolic inhibition. Coupled with its potent insecticidal activity and the potential to harness plant-induced resistance, *N. cadamba* stem bark EO and the active ingredient MeSA presented candidates for sustainable crop protection strategies. The fact that *N. cadamba* is a fast-growing tree [47] further enhanced the feasibility and sustainability of utilizing its stem bark, a by-product of timber production, for this purpose.

## 5. Conclusions

The essential oils of *N. cadamba* exhibit both insecticidal and fungicidal activities. Specifically, the essential oil demonstrated poisonous, knockdown, and fumigant effects against *A. aegypti*, with MeSA identified as the primary active ingredient. Overall, MeSA’s antifungal activity stemmed from its ability to simultaneously target and disrupt multiple cellular components in *R. solani*. These included degradation of the cell wall and plasma membrane, damage to key organelles such as mitochondria, endoplasmic reticulum (ER), vesicle-producing systems (VPS), vesicles, and lomasomes, as well as interference with essential biochemical processes like protein synthesis and respiration. This multi-target disruption led to catastrophic loss of cellular integrity, impaired fungal growth, and ultimately cell death.

## Figures and Tables

**Table 4:** Antioxidant activity of the EOs of *N. cadamba* stem barks and the leaves measured by 2,2-diphenyl-1-picrylhydrazyl (DPPH).

Treatment	Regression Equation	IC_50_ * (mg/mL)	Correlation Coefficient (r)	95% Confidence Limit (mg/mL)
Ascorbic acid	y = 1.75 + 2.46x	0.02	0.98	0.016 − 0.026
EO of the stem barks	y = 1.25 + 1.21x	1.24	0.97	0.88 − 1.90
EO of the leaves	y = 0.32 + 1.33x	3.29	0.97	1.89 − 5.73

Note: *: the concentration of antioxidant required to reduce the DPPH radical concentration by 50%.

**Table 5:** The antifungal activities of the essential oil of stem bark of *N. cadamba* (EC_50_).

Treatment	Fungus	Time	Regression Equation	EC_50_ * (μg/mL)	Correlation Coefficient(r)	95% Confidence Limit (μg/mL)
EO of stem bark	*R. solani*	1 d	y = −6.88 + 4.14x	48.70	0.94	42.80–155.87
	*F. oxysporum*	4 d	y = −7.29 + 2.26x	1229.48	0.96	986.53–1650.02
	*C. gloeosporioides*	4 d	y = −14.27 + 4.81x	928.93	0.98	802.46–1124.78
	*P. grisea*	7 d	y = −7.00 + 2.35x	957.79	0.99	773.38–1241.83
MeSA	*R. solani*	1 d	y = −6.25 + 3.61x	53.91	0.99	32.51–115.21
	*F. oxysporum*	4 d	y = −10.96 + 3.63x	1045.11	0.99	902.83–1234.76
	*C. gloeosporioides*	4 d	y = −13.00 + 4.43x	854.17	0.98	736.06–1032.26
	*P. grisea*	7 d	y = −7.61 + 2.53x	1041.76	0.99	825.37–1265.87
Physcion **^#^**	*R. solani*	1 d	y = −5.98 + 3.04x	93.34	0.97	84.79–103.55

*: EC_50_: median effective concentration; **^#^**: The EC_50_ values of physcion against *F. oxysporum*, *C. gloeosporioides* and *P. grisea* were more than 200 μg/mL.

## Data Availability

The data are contained within the article.

## Supplementary Materials

The following supporting information can be downloaded at https://www.mdpi.com/article/10.3390/plants14233633/s1. Table S1. Chemical components identified in *N. cadamba* leaf essential oil by GC-MS, Figure S1. The antifungal activities of the essential oil of stem bark of *N. cadamba,* MeSA and physcion. References [48,49,50,51,52,53,54,55,56,57,58,59] are cited in the Supplementary Materials.
