# Supplementary material for: Essential Oils from Neolamarckia cadamba: Methyl Salicylate-Rich Stem Bark Oil as a Multi-Functional Biopesticide with Insecticidal and Antifungal Efficacy
Source: Plants (Basel). 2025 Nov 28;14(23):3633. doi: 10.3390/plants14233633 (PMC12694520; doi:10.3390/plants14233633)
Supplement: Supplementary file 1 [file plants-14-03633-s001.zip › plants-3960123-supplementary.pdf]

Table S1. Chemical components identified in *N. cadamba* leaf essential oil by GC-MS

No.	RT	Compounds	Molecular Formula	Percentage (%)	RI_a	RI_b	RI_c	Class	Match	CAS
1	51.78	Phytol	$C_{20}H_{40}O$	23.32	2108	2104[24]	2114	Diterpene	98.81	150-86-7
2	44.25	n-Hexadecanoic acid	$C_{16}H_{32}O_2$	18.41	1970	1961[23]	1968	Fatty acid	97.66	1957/10/3
3	15.10	Methyl salicylate	$C_8H_8O_3$	9.833	1197	1194[20]	1192	Ester	99.48	119-36-8
4	53.33	1-Heneicosene	$C_{21}H_{42}$	8.175	2145	2096[28]	2089.1	Alkene	70.90	1599-68-4
5	24.77	Nerolidol	$C_{15}H_{26}O$	5.33	1559	2053[25]	1564	Sesquiterpene Alcohol	98.50	7212-44-4
6	53.56	Linolenic acid	$C_{18}H_{30}O_2$	4.609	2150	2020[30]	2139	Fatty acid	93.71	463-40-1
7	23.70	(-)-Spathulenol	$C_{15}H_{24}O$	1.609	1528	1599[10]	1577	Sesquiterpene Alcohol	81.75	77171-55-2
8	38.39	Benzyl salicylate	$C_{14}H_{12}O_3$	1.436	1861	1790[31]	1869	Ester	96.70	118-58-1
9	25.15	(*Z*)-3-Hexenyl benzoate	$C_{13}H_{16}O_2$	1.415	1569	2148[32]	1570	Ester	97.30	25152-85-6
10	37.14	Hexahydrofarnesyl acetone	$C_{18}H_{36}O$	1.412	1837	1848[33]	1844	Ketone	97.20	502-69-2

11	25.71	(*E*)-2-Hexenyl benzoate	$C_{13}H_{16}O_2$	1.267	1585	2182[34]	1588	Ester	82.30	76841-70-8
12	25.42	Benzoic acid, hexyl ester	$C_{13}H_{18}O_2$	1.187	1577	1576[30]	1580	Ester	98.13	6789-88-4
13	54.60	Ethyl linolenate	$C_{20}H_{34}O_2$	1.161	2175	2073[30]	2169	Ester	88.87	1191-41-9
14	42.70	Isophytol	$C_{20}H_{40}O$	1.118	1942	1939[28]	1948	Diterpene Alcohol	97.22	505-32-8
15	30.97	1,2-Epoxyhexadecene	$C_{16}H_{32}O$	0.815	1712	-	1708	Epoxide	89.10	7320-37-8
16	21.58	Cabreuva oxide B	$C_{15}H_{24}O$	0.72	1460	1458[21]	1465	Sesquiterpene Epoxide	95.92	107602-53-9
17	26.00	(*E*)-*β*-Farnesene epoxide	$C_{15}H_{24}O$	0.635	1594	1624[35]	1624	Sesquiterpene Epoxide	84.60	83637-40-5
18	53.96	Phytol, acetate	$C_{22}H_{42}O_2$	0.586	2159	2215[33]	2168	Ester	86.20	10236-16-5
19	19.42	Damascenone	$C_{13}H_{18}O$	0.478	1380	1385[23]	1386	Terpenoid Ketone	96.54	23726-93-4
20	57.20	1-Chloroeicosane	$C_{20}H_{41}Cl$	0.476	2298	-	2264	Halogenated Hydrocarbon	73.70	42217-02-7
21	25.02	Widdrol	$C_{15}H_{26}O$	0.458	1566	-	1616.6	Sesquiterpene Alcohol	98.26	6892-80-4

22	29.26	Ledene oxide-(II)	$C_{15}H_{24}O$	0.432	1673	1636[33]	1631	Sesquiterpene Epoxide	76.66	1000159-36-7
23	26.50	Isospathulenol	$C_{15}H_{24}O$	0.404	1607	1628[33]	1640.4	Sesquiterpene Alcohol	82.57	88395-46-4
24	58.21	Tetracosane	$C_{24}H_{50}$	0.358	2398	2400[23]	2400	Alkane	75.47	646-31-1
25	33.34	Benzyl Benzoate	$C_{14}H_{12}O_2$	0.314	1761	1793[48]	1762	Ester	96.40	120-51-4
26	25.83	Tricyclo[4.4.0.0(2,7)]dec-8-ene-3-methanol,α, α,6,8-tetramethyl-stereoisomer	$C_{15}H_{24}O$	0.309	1589	-	1540	Tricyclic Terpene Alcohol	86.55	41370-56-3
27	45.37	Hexadecanoic acid, ethyl ester	$C_{18}H_{36}O_2$	0.268	1991	1992[23]	1993	Ester	95.30	628-97-7
28	42.87	Dibutyl phthalate	$C_{16}H_{22}O_4$	0.267	1945	1964[30]	1965	Ester	92.91	84-74-2
29	41.18	(*Z*)-13-Octadecenal	$C_{18}H_{34}O$	0.264	1914	-	2007	Aldehyde	85.63	58594-45-9
30	24.60	Humulene epoxide II	$C_{15}H_{24}O$	0.249	1554	1610[20]	1579	Sesquiterpene Epoxide	80.60	19888-34-7
31	29.60	*cis*-3-Hexenyl salicylate	$C_{13}H_{16}O_3$	0.244	1681	1584[48]	1670	Ester	88.10	65405-77-8

32	20.51	*α*-Ionone	$C_{13}H_{20}O$	0.227	1422	1427[49]	1426	Terpenoid	96.35	127-41-3
33	56.75	Isopimara-7,15-dien-3-one	$C_{20}H_{30}O$	0.224	2269	-	2257	Diterpene Ketone	86.67	7715-48-2
34	19.56	*(+)*-10-(Acetylmethyl)-3-carene	$C_{13}H_{20}O$	0.21	1386	-	1382	Ester	83.85	163886-28-0
35	50.92	Methyl linolenate	$C_{19}H_{32}O_2$	0.203	2091	2105[10]	2098	Ester	86.94	301-00-8
36	28.49	*β*-Eudesmol	$C_{15}H_{26}O$	0.195	1654	1593[50]	1649	Sesquiterpene Alcohol	87.34	473-15-4
37	37.88	Diisobutyl phthalate	$C_{16}H_{22}O_4$	0.194	1851	1870[10]	1870	Ester	94.69	84-69-5
38	21.10	Geranylacetone	$C_{13}H_{22}O$	0.172	1443	1420[50]	1453	Ketone	87.95	3796-70-1
39	26.78	Humulenol-II	$C_{15}H_{24}O$	0.163	1613	1608[10]	1650.4	Sesquiterpene Alcohol	85.50	19888-00-7
40	27.54	*γ*-Eudesmol	$C_{15}H_{26}O$	0.151	1631	1633[20]	1631	Sesquiterpene Alcohol	83.52	1209-71-8
41	55.87	3,7,11,15-Tetramethylhexadec-2-en-1-yl acetate	$C_{22}H_{42}O_2$	0.146	2212	-	2232	Ester	87.62	76337-16-1

42	57.71	3-Ethyl-5-(2-ethyl-butyl) octadecane	$C_{26}H_{54}$	0.143	2348	2589[51]	2413	Alkane	62.00	55282-12-7
43	36.92	Neophytadiene	$C_{20}H_{38}$	0.137	1833	1844[52]	1840.6	Diterpene Hydrocarbon	94.50	504-96-1
44	12.69	Linalool	$C_{10}H_{18}O$	0.125	1099	1097[20]	1099	Monoterpene Alcohol	99.07	78-70-6
45	39.43	*Z*-(13,14-Epoxy)-tetradec-11-en-1-ol acetate	$C_{16}H_{28}O_3$	0.113	1881	-	1849	Ester	79.70	
46	55.64	Docosane	$C_{22}H_{46}$	0.113	2199	2200[10]	2200	Alkane	82.59	629-97-0
47	54.40	Ethyl Linoleate	$C_{20}H_{36}O_2$	0.11	2170	2144[33]	2162	Ester	93.57	544-35-4
48	33.06	Tetradecanoic acid	$C_{14}H_{28}O_2$	0.108	1755	1764[10]	1768	Fatty acid	86.41	544-63-8
49	18.90	1,2-Dihydro-1,5,8-tri-methyl-naphthalene	$C_{13}H_{16}$	0.106	1358	1299[51]	1354	Aromatic Hydrocarbon	90.50	4506-36-9
50	46.79	3,7,11,15-Tetramethyl-hexadeca-1,6,10,14-tetraen-3-ol	$C_{20}H_{34}O$	0.106	2016	-	2046	Diterpene Alcohol	87.41	68931-30-6

51	47.53	Kaur-16-ene	$C_{20}H_{32}$	0.104	2030	2041[30]	2041	Diterpene Hydrocarbon	93.93	562-28-7
52	21.69	Cabreuva oxide C	$C_{15}H_{24}O$	0.102	1464	1468[53]	1469	Sesquiterpene Epoxide	88.21	107602-55-1
53	45.20	5-Octadecenal	$C_{18}H_{34}O$	0.094	1987	-	2002.4	Aldehyde	75.20	56554-88-2
54	17.95	2,4-Decadienal, (*E*, *E*)-	$C_{10}H_{16}O$	0.091	1318	1315[28]	1317	Aldehyde	91.16	25152-84-5
55	39.68	Methyl hexadeca-4,7,10,13-tetraenoate	$C_{17}H_{26}O_2$	0.09	1886	-	1910	Ester	83.30	1417890-65-3
56	41.59	Methyl palmitate	$C_{17}H_{34}O_2$	0.084	1921	1928[21]	1926	Ester	94.07	112-39-0
57	57.04	Larixol	$C_{20}H_{34}O_2$	0.078	2288	2211[10]	2265	Diterpene Alcohol	79.80	1438-66-0
58	37.57	Tetradecanoic acid, trimethylslyl ester	$C_{17}H_{36}O_2Si$	0.07	1845	1862[21]	1850	Ester	87.13	18603-17-3
59	22.45	1,11-Oxido-calamenene	$C_{15}H_{20}O$	0.068	1490	1511[48]	1493	Sesquiterpene Epoxide	90.10	143785-42-6
60	57.83	10,13-Eicosadienoic acid,	$C_{21}H_{38}O_2$	0.067	2360	-	2292	Ester	74.90	30223-50-8

		methyl ester								
61	57.49	*cis*-13-Eicosenoic acid	$C_{20}H_{38}O_2$	0.066	2327	-	2365.5	Fatty Acid	77.10	17735-94-3
62	16.85	Ethyl salicylate	$C_9H_{10}O_3$	0.064	1271	-	1269	Ester	90.43	118-61-6
63	30.55	(*Z*)-*α*-trans-Bergamotol	$C_{15}H_{24}O$	0.064	1703	2247[32]	1690	Sesquiterpene Alcohol	78.10	88034-74-6
64	21.50	2,6,10-Trimethyl-tridecane	$C_{16}H_{34}$	0.062	1457	1462[22]	1449	Alkane	90.46	3891-99-4
65	24.25	*cis*-*Z*-*α*-Bisabolene epoxide	$C_{15}H_{24}O$	0.062	1544	-	1531	Sesquiterpene Epoxide	77.10	121467-35-4
66	29.79	*α*-Bisabolol	$C_{15}H_{26}O$	0.062	1685	1917[26]	1684	Sesquiterpene Alcohol	75.24	515-69-5
67	31.32	Aromadendrene oxide-(1)	$C_{15}H_{24}O$	0.062	1719	1631[33]	1672	Sesquiterpene Epoxide	81.09	85760-81-2
68	23.26	1, 8-(2H,5H)- Naphthalenedione, hexahydro-8a-methyl, *cis*	$C_{11}H_{16}O_2$	0.06	1515	1523[23]	1517	Diketone	76.30	83406-41-1
69	40.65	Farnesyl acetone	$C_{18}H_{30}O$	0.059	1904	1912[21]	1919	Ketone	89.02	1117-52-8

70	16.33	Geraniol	$C_{10}H_{18}O$	0.057	1249	1254[20]	1255	Monoterpene Alcohol	93.35	106-24-1
71	31.93	(*E*, *E*)-Farnesal	$C_{15}H_{24}O$	0.056	1732	1748[27]	1735	Terpenoid Aldehyde	74.43	502-67-0
72	19.26	2,6,10-Trimethyldodecane	$C_{15}H_{32}$	0.054	1373	-	1379	Alkane	91.90	3891-98-3
73	22.21	*β*-Ionone epoxide	$C_{13}H_{20}O_2$	0.052	1482	-	1473	Terpenoid Epoxide	84.70	23267-57-4
74	48.30	Trimethylsilyl palmitoate	$C_{19}H_{40}O_2Si$	0.051	2044	-	2050	Ester	84.76	55520-89-3
75	57.65	5-Methyl-5-(4,8,12-trimethyl-tridecyl) Dihydro-2(3H)-furanone	$C_{21}H_{40}O_2$	0.051	2343	-	2364	Lactone	79.55	96168-15-9
76	27.78	Caryophylla-4(12),8(13)-dien-5-*α*-ol	$C_{15}H_{24}O$	0.05	1637	1641[20]	1640.2	Sesquiterpene Alco-hol	82.40	19431-79-9
77	58.00	2-(*Z*)-9-Octa-decenyloxy-ethanol	$C_{20}H_{40}O_2$	0.049	2377	2336[50]	2336	Fatty Ether	76.70	5353-25-3
78	24.48	Limonen-6-ol, pivalate	$C_{15}H_{24}O_2$	0.048	1550	-	1560	Ester	74.40	
79	6.85	*cis*-3-Hexen-1-ol	$C_6H_{12}O$	0.047	851	849[23]	857	Alcohol	93.99	928-96-1
80	30.18	2-Pentadecanone	$C_{15}H_{30}O$	0.047	1694	1699[52]	1698	Ketone	78.45	2345-28-0
81	13.52	4-Acetyl-1-methyl-cyclo-hexene	$C_9H_{14}O$	0.046	1133	1118[29]	1137	Ketone	95.32	6090-9-1

82	30.68	ent-Germacra-4(15),5,10(14)-trien-1*β*-ol	$C_{15}H_{24}O$	0.044	1706	1675[24]	1694.5	Sesquiterpene Alcohol	77.80	81968-62-9
83	20.98	1-Oxaspiro [2.5] octane, 5,5-dimethyl- 4-(3-methyl- 1,3-butadienyl)-	$C_{14}H_{22}O$	0.043	1439	1768[54]	1431	Spirocyclic Ether	77.60	925919-79-5
84	49.07	12-Methyl-*E*, *E*-2,13-octadecadien-1-ol	$C_{19}H_{36}O$	0.043	2057	-	2104	Unsaturated Fatty Alcohol	80.00	874197-21-4
85	19.00	2-Undecenal	$C_{11}H_{20}O$	0.042	1362	1397[48]	1367	Aldehyde	91.12	2463-77-6
86	12.81	Nonanal	$C_9H_{18}O$	0.041	1104	1103[20]	1104	Aldehyde	94.76	124-19-6
87	26.96	Caryophyllene oxide	$C_{15}H_{24}O$	0.041	1618	1577[33]	1593	Sesquiterpene Epoxide	81.70	1139-30-6
88	20.32	Ionone	$C_{13}H_{20}O$	0.04	1415	-	1426	Terpenoid Ketone	75.50	8013-90-9
89	14.38	1,3,4-Trimethyl,3-cyclohexene-1-carboxaldehyde	$C_{10}H_{16}O$	0.039	1168	1771[55]	1171	Aldehyde	71.40	40702-26-9
90	20.93	3-Methylbutyl butanoate	$C_{12}H_{16}O_2$	0.038	1437	1266[56]	1439	Ester	85.97	94-46-2
91	50.64	Methyl linoleate	$C_{19}H_{34}O_2$	0.037	2086	2099[10]	2092	Ester	73.51	112-63-0

92	56.53	Kaurenal	$C_{20}H_{30}O$	0.037	2255	-	2303	Diterpene Aldehyde	75.92	14046-84-5
93	11.13	Lavender lactone	$C_7H_{10}O_2$	0.036	1037	1044[48]	1043	Ester	97.49	1073-11-6
94	57.77	Kaurenol	$C_{20}H_{32}O$	0.036	2354	-	2400	Diterpene Alcohol	83.14	2300-11-0
95	20.45	2H-Cyclopropa[g] benzo-furan, 4,5,5a,6,6a,6b-hexahydro-4,4,6b-trimethyl-2-(1-methylethenyl)-	$C_{15}H_{22}O$	0.035	1420	1567[26]	1438	Terpenoid	81.10	102681-49-2
96	22.56	Shyobunone	$C_{15}H_{24}O$	0.035	1494	1456[32]	1518.9	Sesquiterpene Ketone	74.80	21698-44-2
97	28.64	Neointermedeol	$C_{15}H_{26}O$	0.035	1658	1660[35]	1660	Sesquiterpene Alcohol	86.38	5945-72-2
98	36.57	Farnesoic acid	$C_{15}H_{24}O_2$	0.035	1826	-	1822	Terpenoid Carboxylic Acid	76.37	7548-13-2
99	5.82	Caproaldehyde	$C_6H_{12}O$	0.034	800	-	800	Aldehyde	93.76	108-38-3
100	16.64	(*E*)-2-Decenal	$C_{10}H_{18}O$	0.033	1262	1266[23]	1263	Aldehyde	94.07	3913-81-3

101	23.44	1H-Inden-1-one, 2,3-dihydro-3,3,4,6-tetramethyl-	$C_{13}H_{16}O$	0.033	1520	-	1579	Ketone	68.20	55255-42-0
102	7.10	1-Hexanol	$C_6H_{14}O$	0.032	864	864[23]	868	Alcohol	94.69	111-27-3
103	15.30	Decanal	$C_{10}H_{20}O$	0.032	1205	1204[50]	1206	Aldehyde	95.72	112-31-2
104	40.90	Cyclopentadecanone	$C_{15}H_{28}O$	0.032	1909	-	1861	Ketone	73.19	502-72-7
105	7.03	*cis*-2-Hexen-1-ol	$C_6H_{12}O$	0.03	861	-	868	Alcohol	93.17	928-94-9
106	14.47	Benzoic acid, ethyl ester	$C_9H_{10}O_2$	0.03	1171	1180[57]	1171	Ester	91.01	93-89-0
107	33.94	9-methylene-9H-Fluorene	$C_{14}H_{10}$	0.03	1774	-	1711	Aromatic Hydrocar-bon	77.32	4425-82-5
108	15.71	*β*-Cyclocitral	$C_{10}H_{16}O$	0.029	1223	1221[21]	1220	Terpenoid Aldehyde	86.86	432-25-7
109	32.04	Oxacyclododeca-6,9-dien-2-one, 7-methyl-, (Z,E)- (9CI)	$C_{12}H_{18}O_2$	0.028	1734	-	1718	Lactone	69.31	70968-72-8
110	35.90	Hexadecanal	$C_{16}H_{32}O$	0.028	1813	1821[23]	1817	Aldehyde	80.93	629-80-1
111	10.49	(*E*, *E*)-2,4-Heptadienal	$C_7H_{10}O$	0.025	1011	1015[21]	1012	Aldehyde	90.08	4313-03-5
112	8.92	5.5-dimethyl-2 (5H) -furanone	$C_6H_8O_2$	0.024	946	-	952	Lactone	94.00	20019-64-1

113	15.41	Neodihydrocarveol	$C_{10}H_{18}O$	0.024	1210	-	1232.2	Monoterpene Alcohol	75.50	18675-33-7
114	19.92	Tetrahydrogeranyl acetone	$C_{13}H_{26}O$	0.023	1401	1407[58]	1408	Ketone	72.40	1604-34-8
115	41.03	3-Methyl-2-(3,7,11-trimethyl-dodecyl) furan	$C_{20}H_{36}O$	0.023	1911	-	1931	Furan	58.56	166773-55-3
116	45.78	Eicosane	$C_{20}H_{42}$	0.023	1998	1998[25]	2000	Alkane	69.03	112-95-8
117	47.02	1-Methylethyl hexadecanoate	$C_{19}H_{38}O_2$	0.022	2021	2226[32]	2023	Ester	77.30	142-91-6
118	47.28	Verimol K	$C_{14}H_{12}O_4$	0.022	2025	-	2053	Lactone	65.48	85985-75-7
119	38.20	10-Octadecenal	$C_{18}H_{34}O$	0.021	1857	-	1863	Fatty Aldehyde	72.10	56554-92-8
120	35.12	Octadecane	$C_{18}H_{38}$	0.018	1798	1800[31]	1800	Alkane	69.28	593-45-3
121	42.38	*cis*-7-Hexadecenoic acid	$C_{16}H_{30}O_2$	0.018	1936	-	1961.4	Fatty acid	71.90	2416-19-5
122	10.28	(*E*)-3-Hexen-1-yl acetate	$C_8H_{14}O_2$	0.016	1003	-	1005	Ester	89.14	3681-82-1
123	34.95	(*Z*)-7-Hexadecenal	$C_{16}H_{34}O$	0.016	1795	-	1798	Aldehyde	88.90	56797-40-1
124	11.49	*cis*-Arbusculone	$C_9H_{14}O_2$	0.015	1051	1052[59]	1052	Sesquiterpene Ketone	86.51	56469-37-5
125	56.17	16 *β* H-Kauran-16-ol	$C_{20}H_{34}O$	0.015	2232	-	2266	Diterpene Alcohol	70.17	5354-44-9

126	16.76	Citral	$C_{10}H_{16}O$	0.013	1267	-	1276	Monoterpene Aldehyde	89.41	5392-40-5
127	39.88	7-Methyl-Z-tetradecen-1-ol acetate	$C_{17}H_{32}O_2$	0.013	1890	-	1822	Ester	61.70	1000130-99-6
128	45.62	*tert*-Hexadecanethiol	$C_{16}H_{34}S$	0.013	1995	-	1831	Thiol	61.67	25360-09-2
129	11.62	1-(1,2-dimethyl-cyclopent-2-enyl)-ethanone	$C_9H_{14}O$	0.012	1056	-	1041	Ketone	66.51	70987-82-5
130	14.19	(*E*)-2-Nonenal	$C_9H_{16}O$	0.012	1160	1164[23]	1162	Aldehyde	88.53	18829-56-6
131	32.31	2,5,5,8a-Tetramethyl-4-methylene-6,7,8,8a-tetrahydro-4H,5H-chromen-4a-yl hydroperoxide	$C_{14}H_{22}O_3$	0.012	1740	1740[10]	1771	Hydroperoxide	69.93	1000192-73-0
132	56.46	*cis*-9,10-Epoxystearic acid	$C_{18}H_{34}O_3$	0.012	2250	-	2219	Epoxy Fatty Acid	76.10	24560-98-3
			**Total**	**93.94**						

Note: RT, the retention time; RIa, the Kovats retention index of each component calculated by NIST software and ion spectrum of C7-C30 n-alkane mixture; RIb, the retention index in NIST 17 mass spectrometry library; RIc, the retention index in the literature.

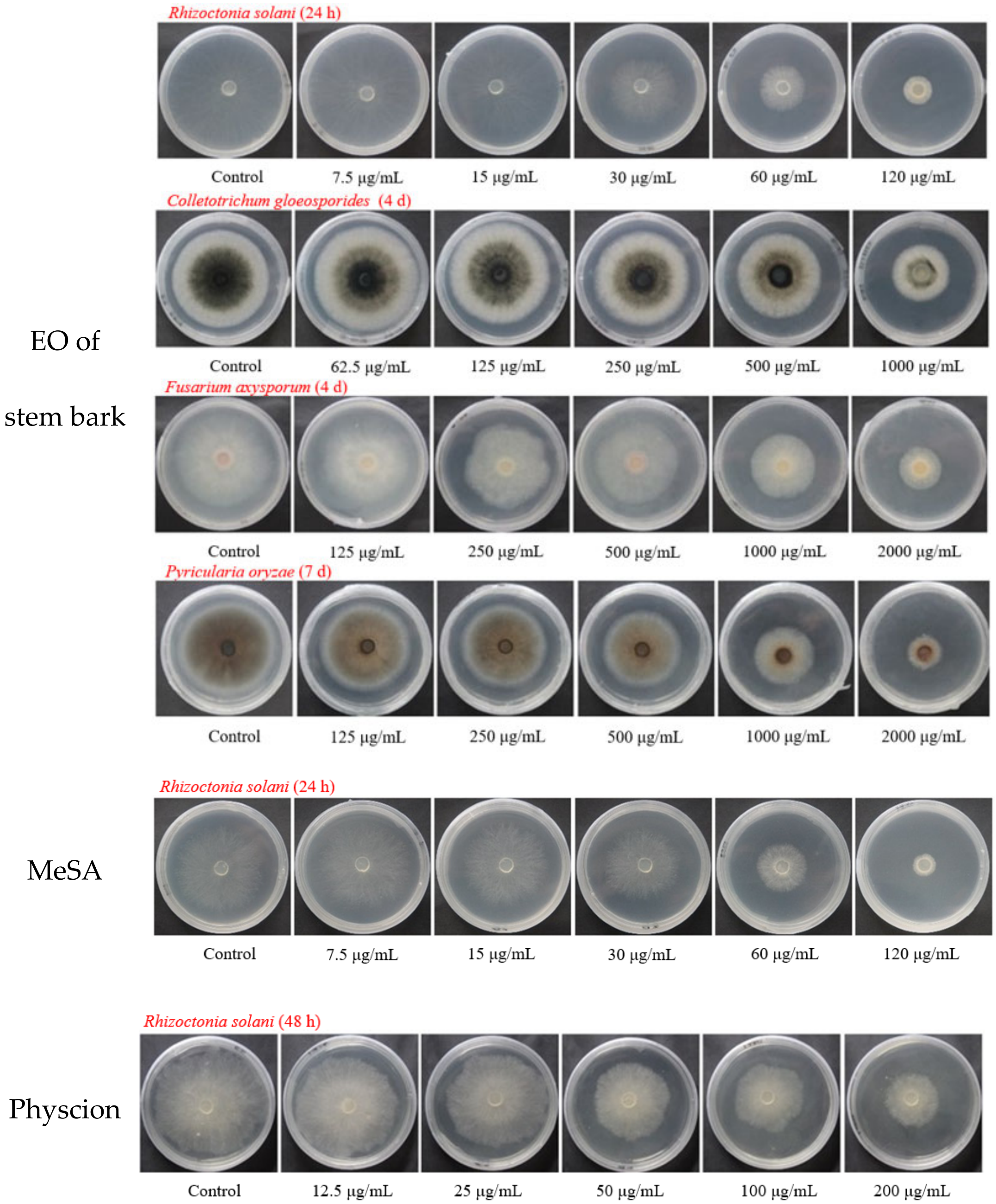

Figure S1. The antifungal activities of the essential oil of stem bark of *N. cadamba,* MeSA and physcion.
